# Can You Determine the Cause of This Patient’s Vision Changes?

**DOI:** 10.6004/jadpro.2012.3.6.10

**Published:** 2012-11-01

**Authors:** Patricia Palmer,, Megan Nolan

**Affiliations:** From University of California–Davis Medical Center, Sacramento, California

## ABSTRACT

Mrs. G. is a post–stem cell transplant patient who was admitted to the hospital with a presumed lower GI bleed in the setting of chronic thrombocytopenia and graft-vs.-host disease. On hospital day 21, she has a mild headache, fever, incoordination, neck stiffness, and significant vision changes. View the MRI results and see if you can choose the correct diagnosis.

**Can You Determine the Cause of This Patient’s Vision Changes?:**
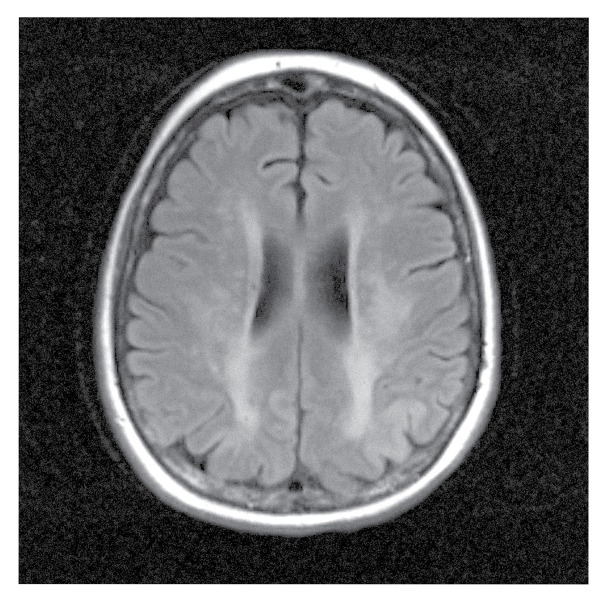


## History

Mrs. G., a 56-year-old woman with a history of osteoporosis, migraine headaches, and active tobacco usage, was diagnosed with myelodysplasia in November 2009. In August 2010, she underwent related-donor allogeneic stem cell transplantation. Due to a suboptimal CD34 donor harvest, only white blood cells engrafted well. Both anemia and thrombocytopenia persisted after transplant, leaving her transfusion-dependent. In March 2011, GI tract graft-vs.-host disease (GVHD) developed, causing large volumes of watery diarrhea. The GVHD resolved after steroids were added to her cyclosporine GVHD prophylaxis.

In February 2012, Mrs. G. presented with abdominal pain and bloody stools and was admitted for a presumed lower GI bleed in the setting of chronic thrombocytopenia. Colonoscopy revealed grade 1 GVHD, which was treated with resumption of steroids and continued cyclosporine. Mrs. G.’s hospital course was complicated by urosepsis, as well as the fact that she became refractory to standard platelet transfusions and required human lymphocyte antigen–matched platelets daily.

## Chief Complaint

On March 1, 2012 (hospital day 21), Mrs. G. reported a mild headache (5/10) and spiked a temperature of 39.1°C (102.4°F) after transfusion of two units of packed red blood cells. Later that day, she called her nurse into her room for assistance to the commode, but was unable to transfer safely due to her incoordination; she was incontinent and complained of neck stiffness and vision changes (seeing only shadows).

**Physical Examination and Diagnostic Studies** 

On physical examination, Mrs. G.’s temperature was 38.1°C (100.6°F), heart rate 101, respirations 14, blood pressure 113/67, and O_2_ saturation 92% on 2 L nasal cannula. She was alert and oriented, with brief periods of confusion. She had intact pupillary responses and was able to perceive light and shadows, but unable to count fingers placed directly in front of her face. Laboratory results from 7 a.m. were as follows: white blood cells 1.5 k/mm, hemoglobin 10.6 g/dL, hematocrit 31.8%, and platelets 6,000/mm3. A STAT head CT was performed, followed by an MRI. Ophthalmology reported grossly normal fundoscopic exam with a single microaneurysm in the far periphery of the right eye, thought to be secondary to thrombocytopenia. Neurology reported negative Kernig and Brudzinski signs, and intact cranial nerves. Deep-tendon reflexes were 3+ in bilateral upper and lower extremities; strength and sensation were normal. MRI demonstrated subcortical and cortical edema, more pronounced in the bilateral parietal lobes (see Fig. 1).

**Choose the correct diagnosis:** 

- POSTERIOR REVERSIBLE ENCEPHALOPATHY SYNDROME
- ISCHEMIC STROKE
- SPONTANEOUS CEREBRAL HEMORRHAGE
- Scroll down for correct answer.

**Can You Determine the Cause of This Patient’s Vision Changes?:**
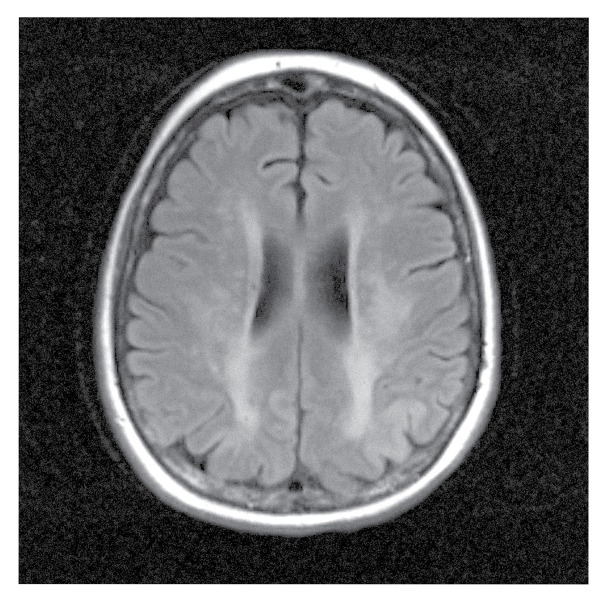


**Posterior reversible encephalopathy syndrome (PRES)** is a constellation of symptoms including headache, seizure, altered mental status, visual disturbances such as cortical blindness, and MRI findings that were first formally described by Hinchey et al. in 1996. Cortical blindness is a partial or full loss of sight in the absence of abnormalities upon ophthalmic examination, resulting from damage to the cerebral visual pathways (Leveque, Trobe, & Sokol, 2012). Other potential symptoms include brisk tendon reflexes and weakness or incoordination of the limbs (Feske, 2011). Classic MRI findings are predominantly posterior (parieto-occipital) white matter edema found in both hemispheres of the cerebral cortex, but other areas may be affected as well (Hinchey et al., 1996). MRI abnormalities are distinguished from ischemia and infarction by potential causative factors and the utilization of both diffusion-weighted imaging (DWI) and T2-weighted fluid-attenuated inversion recovery (FLAIR; Doelken et al., 2007; Pula & Eggenberger, 2008; Feske, 2011). In the case of PRES, DWI sequences are normal, but T2-weighted FLAIR images (Fig. 1) will reveal bilateral subcortical edema, suggesting a vasogenic, rather than ischemic, cause of edema (Feske, 2011). Clinical correlation is warranted, but imaging with MRI can help the clinician determine the cause of the patient’s encephalopathic symptoms. In this case, the MRI findings were suggestive of PRES when correlated with the clinical setting.

Encephalopathy due to reversible cerebral edema is an important cause of neurologic morbidity seen in many disorders including eclampsia, renal disease, acute hypertension, sepsis, and exposure to immunosuppressants such as cyclosporine (Fugate et al., 2010). While the exact mechanism of pathogenesis remains unknown, available theories focus on the development of vasogenic cerebral edema as a result of either increased capillary filtration pressure or relative failure of the blood-brain barrier. Cyclosporine is routinely utilized in stem cell transplant recipients as GVHD prophylaxis, and neurotoxicity such as confusion, visual disturbances, ataxia, and seizures have been reported in approximately 10% of patients (Schwartz et al., 1995). Cyclosporine neurotoxicity is thought to be a result of endothelial damage in the brain, making cyclosporine toxicity a component of PRES. Interestingly, cyclosporine neurotoxicity can occur at both elevated and normal (therapeutic) blood levels (Erbetta et al., 2008; Torelli et al., 2011).

## Explanation of Incorrect Answers

**Ischemic stroke** is suspected whenever a patient is found to have a focal neurologic deficit and requires urgent assessment and treatment to prevent brain loss (Weinhardt & Jacobson, 2012). The symptoms typically seen with a stroke are unilateral facial and/or extremity numbness and/or weakness, difficulty speaking or understanding speech, difficulty with vision, loss of balance or coordination, severe unexplained headache, and/or unexplained nausea and vomiting. The best-studied scales for rapid diagnosis of stroke are the Face Arm Speech Test (FAST), the Cincinnati Prehospital Stroke Scale (CPSS), the Los Angeles Prehospital Stroke Screen (LAPSS), and the Recognition of Stroke in the Emergency Room (ROSIER; Dawson & Walters, 2005). In the case of Mrs. G., an urgent CT scan and MRI along with neurology and ophthalmology consultations were ordered. While the MRI did show a small area of hemorrhage, it did not explain the sudden blindness. On neurologic exam she had two focal findings: blindness and the inability to ambulate, but her weakness was bilateral. Using the FAST scale, Mrs. G. would have scored negative for stroke.

**Spontaneous cerebral hemorrhage.** Intracerebral hemorrhage (ICH) is the second most common cause of stroke (Broderick et al., 1993). In a patient with a chronically low platelet count, there is always a concern for spontaneous bleeding. In the setting of ICH, the primary mechanisms of brain injury are direct mechanical injury to the brain parenchyma or increased intracranial pressure and herniation (Xu et al., 1993). Mrs. G.’s platelet count was 6,000 on the morning of this event and indeed her emergent CT scan did show a small intracerebral bleed, but the location of the bleed did not explain her sudden blindness.

## Treatment

Since its first formal description in 1996, PRES has been described in both case studies and retrospective reports, but continues to be relatively poorly understood. Focus remains on reducing hypertension, if present, and removing potential causative agents.

Mrs. G.’s treatment included continuing to transfuse HLA-matched platelets to counteract potentially devastating effects of profound thrombocytopenia, stopping cyclosporine, and closely monitoring blood pressure. Hypertension and seizures are often presenting symptoms of PRES (Feske, 2011). Treatment is aimed at the underlying cause, and pharmacologic management of hypertension and seizures is warranted. In addition, cessation of cyclosporine or dose reduction is recommended (Pula & Eggenberger, 2008; Feske, 2011). Early recognition and treatment of the symptoms associated with hypertensive encephalopathy and immunosuppressant-related neurotoxicity are key in the reversibility of PRES. Reimaging of the patient after resolution of symptoms should reveal a complete or partial resolution of abnormalities, reinforcing the diagnosis of PRES (Pula & Eggenberger, 2008). Mrs. G. experienced rapid improvement of her vision and coordination after the removal of cyclosporine.

## Follow-up

Mrs. G. rapidly regained coordination of her extremities and sight in her right eye after cessation of the cyclosporine but continues to experience some blurriness in her left eye. She was able to be discharged from the hospital after a second infusion of plerixafor (Mozobil)-mobilized stem cells from her original donor and is currently being followed as an outpatient; she continues to require weekly platelet transfusions.
